# Joint global and local interpretation method for CIN status classification in breast cancer

**DOI:** 10.1016/j.heliyon.2024.e27054

**Published:** 2024-02-28

**Authors:** Liangliang Liu, Pei Zhang, Zhihong Liu, Tong Sun, Hongbo Qiao

**Affiliations:** College of Information and Management Science, Henan Agricultural University, Zhengzhou, Henan 450046, PR China

**Keywords:** Chromosomal instability, Breast cancer, XGBoost, SHAP, miRNAs

## Abstract

Breast cancer is among the cancer types with the highest numbers of new cases. The study of this disease from a microscopic perspective has been a prominent research topic. Previous studies have shown that microRNAs (miRNAs) are closely linked to chromosomal instability (CIN). Correctly predicting CIN status from miRNAs can help to improve the survival of breast cancer patients. In this study, a joint global and local interpretation method called GL_XGBoost is proposed for predicting CIN status in breast cancer. GL_XGBoost integrates the eXtreme Gradient Boosting (XGBoost) and SHapley Additive exPlanation (SHAP) methods. XGBoost is used to predict CIN status from miRNA data, whereas SHAP is used to select miRNA features that have strong relationships with CIN. Furthermore, SHAP's rich visualization strategies enhance the interpretability of the entire model at the global and local levels. The performance of GL_XGBoost is validated on the TCGA-BRCA dataset, and it is shown to have an accuracy of 78.57% and an area under the curve value of 0.87. Rich visual analysis is used to explain the relationships between miRNAs and CIN status from different perspectives. Our study demonstrates an intuitive way of exploring the relationship between CIN and cancer from a microscopic perspective.

## 1 Introduction

Breast cancer has emerged as one of the most prevalent cancers among women worldwide and has become the leading cause of death among female cancer patients. According to the World Health Organization, an estimated 685,000 women succumbed to breast cancer in 2020; thus, breast cancer accounted for 16% of all female cancer-related mortalities, equivalent to one of every six women who died from cancer [1]. Extensive clinical investigations have established a strong correlation between the survival outcomes of breast cancer patients and chromosomal instability (CIN) [2], [3], [4], [5]. CIN is the sustained occurrence of chromosomal alterations, including both structural and numerical anomalies such as deletions, duplications, breaks, and translocations [5]. CIN serves as the foundation for numerous diseases but has a particular impact on tumors, underlying a significant portion of the intra-tumor heterogeneity observed in cancer and exerting a profound influence on the clinical management and treatment of cancer patients [5], [6]. Empirical evidence from numerous studies has confirmed that cancer patients with high CIN typically have poor prognosis and shorter-term survival [7], [8], [9]. Therefore, correctly classifying the CIN status is crucial for predicting the prognosis of cancer patients.

Clinical studies have provided compelling evidence that microRNAs (miRNAs) are highly relevant to CIN [10]. miRNAs are a class of non-coding small RNA molecules, typically ranging from 20 to 24 nucleotides in length. They regulate gene expression by binding to their target mRNA [11] and have an important role in post-transcriptional gene regulation. Dysregulation of miRNAs has been implicated in a multitude of diseases, including cancer. Notably, miRNAs have also been shown to regulate the expression of genes associated with centrosomal replication, a process of paramount importance in ensuring the accurate segregation of chromosomes during cell division. For instance, *miR-24*, *miR-34*, and *miR-421* have been shown to regulate the expression of pivotal genes involved in the DNA repair process [12], [13], [14], [15], [16]. Moreover, CIN can affect the expression and functionality of miRNAs, which in turn affects their binding and regulatory effects on target genes. In summary, miRNAs and CIN exhibit a close interrelationship, mutually influencing each other and contributing to the occurrence and progression of cancer.

## 2 Related work

To better predict the survival of breast cancer patients, growing numbers of clinicians and biologists are studying breast cancer disease from a microscopic perspective. Their studies have strongly implicated CIN in the prognosis of breast cancer patients. Smid et al. [17] demonstrated that CIN was involved in the progression of multiple breast cancer subtypes, including estrogen receptor-positive, luminal B and *HER2/neu*. Carter et al. [18] studied CIN based on a number of specific genes. They found a direct correlation between net overexpression of CIN and unfavorable prognostic outcomes in a dataset of 12 cancers representing six cancer types. This study further revealed a significant prognostic link between gene expression profiles associated with CIN and breast cancer metastasis. Moreover McClelland [19] and Vargas-Rondón et al. [20] found a noteworthy association between CIN and higher tumor grades, reduced survival rates, and increased cancer recurrence. Intra-tumor heterogeneity mediated through CIN is also associated with elevated risks of patient mortality or recurrence. In summary, breast cancer patients with high CIN tend to have poorer response rates and higher resistance to treatment modalities such as radiotherapy and chemotherapy. Consequently, these patients face greater challenges in treatment and experience higher recurrence rates. Similar associations have been verified in other cancer types, including lung, gastric, and colorectal cancers [21], [22], [23]. Furthermore, researchers have identified crucial roles of miRNAs in tumorigenesis and in tumor progression and treatment [24]. However, the aforementioned studies addressing prognosis in breast cancer mainly used biomedical experimental analysis methods, which rely on the subjective experience of researchers and require substantial human and material resources to yield predictive outcomes. Moreover, these studies were constrained by limited sample sizes, making it difficult to intuitively demonstrate correlations between CIN status and miRNA molecules. Hence, validation on a large amount of data is warranted to provide a more definitive understanding of this relationship.

With advances in computer technology, artificial intelligence methods such as machine learning and deep learning are being applied to mine hidden but relevant information about diseases and aid clinical diagnosis. For example, Wu et al. [25] employed four machine learning methods to distinguish triple-negative breast cancer from non-triple-negative breast cancer using gene expression data and clinical information. They found that support vector machines (SVMs) outperformed the other three methods. Podolsky et al. [26] evaluated various machine learning algorithms for classification of lung cancer based on gene expression levels, demonstrating the effectiveness of machine learning approaches on diverse datasets. Plaimas et al. [27] developed a machine learning technique to predict the essential genes in a bacterial metabolic network with good accuracy. Machine learning has many applications in different classification and prediction problems; however, its models are susceptible to overfitting and performance improvement is necessary. By contrast, deep learning has exhibited superior performance in the medical field [28], [29]. For example, Ahmed et al. [30] applied an improved deep neural network to gene expression data for classification and prediction. It achieved classification accuracies higher than 91% across all four cancers and obtained the highest accuracy of 99% on the leukemia dataset. However, the lack of interpretability associated with deep learning methods restricts their applications in clinical diagnosis. Therefore, there is an urgent need to develop an interpretable model that can accurately predict the relationships between miRNAs and CIN status, to provide valuable technical support for prognostic assessment of breast cancer patients from a microscopic perspective.

Some high-performance and interpretable ensemble learning methods, including random forest (RF), adaptive boosting (AdaBoost), and XGBoost, have been applied in disease diagnosis. Taylor et al. [31] used several machine learning methods to predict urinary tract infection conditions. The experimental results showed that ensemble methods significantly improved the specificity and sensitivity of urinary tract infection prediction on both the whole and the reduced datasets. XGBoost achieved the best performance (area under the curve [AUC] = 0.826–0.904), outperforming RF, AdaBoost, and SVM models. Iwase et al. [32] used machine learning and logistic regression methods to investigate the mortality rate of patients in intensive care units (ICUs). Among the three machine learning methods, RF and XGBoost showed similar predictive ability regarding ICU mortality rates in the test queue, achieving AUC values exceeding 94%. They also selected key variables that could contribute to accurate prediction based on the RF method, helping to improve treatment efficiency. Devan and Khare et al. [33] first used XGBoost for feature selection when solving the problem of network intrusion classification and selected important features by calculating feature importance scores at the global level. Then they combined this with deep neural networks for classification and achieved better classification results. These findings show that XGBoost is capable of handling high-dimensional data and exhibits good robustness in the presence of missing values and outliers. However, it should be noted that XGBoost has three feature selection strategies, which are calculated in inconsistent ways, and it cannot explain the model's prediction process at an individual sample level.

In this study, to address the above issues, we propose a novel approach, GL_XGBoost, for breast cancer CIN status classification. Our method combines a joint global and local interpretation of the implicit relationships between miRNA features and the CIN status of breast cancer patients. In addition, we incorporate visual analysis methods to enhance the interpretability of the model. The proposed approach aims to provide insight and guidance that will be of use in the treatment and prognosis of breast cancer patients. Our major contributions are as follows.

(1) We propose a novel interpretable ensemble method for predicting CIN status in breast cancer.
(2) We adopt the concepts of Shapley value theory and game theory to select important miRNA features and analyze the prediction process of the XGBoost model.
(3) We used various visualizations to illustrate the correlations between miRNA features and the CIN status in breast cancer.

## 3 Methods

### 3.1 The proposed method

**Figure 1:**
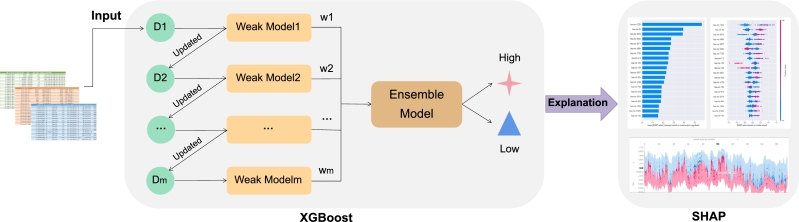
The architecture of GL_XGBoost.

Model interpretability refers to how well the reasoning behind a model's predictions or decision-making process can be comprehended and explained. In clinical diagnosis, an interpretable auxiliary diagnostic model can help physicians and patients to understand the logical progression of the diagnostic process. In the present study, we propose an ensemble method, GL_XGBoost, consisting of the XGBoost and SHAP methods. This ensemble method is intended to predict CIN status from miRNA sequencing data of breast cancer patients. Fig. 1 shows the architecture of our proposed method.

As shown in Fig. 1, our proposed method consists of three parts: the input data, the classifier module, and the interpretation module. We use clinical information, CNV data, and miRNA sequencing data as the input data. As our classified, we employ XGBoost, which combine weak classifiers (e.g., $WeakModel_{1}$–$WeakModel_{m}$ in Fig. 1) to create a strong classifier (e.g., the ensemble model in Fig. 1) [34]. In XGBoost, $D_{1}$ is the pre-processed dataset, whereas $D_{2}$ to $D_{m}$ are new datasets trained using previous weak classifiers and continuously updated sample weights, respectively. Each weak model corresponds to a weak classifier within XGBoost, with $w_{i}$ representing the weight of a weak model. During the model training process, the weights of the incorrectly predicted samples in the $weakmodel_{i-1}$ will be improved in the subsequent classifier ($weakmodel_{i}$), whereas the weights of the correctly predicted samples will be reduced. After a new round of weight updates, the $weakmodel_{i}$ will place greater emphasis on samples that were previously predicted incorrectly by the $weakmodel_{i-1}$. Then, based on the classification results of each weak model, different weights are assigned to combine the models and form a strong classifier [35]. SHAP functions as an interpretable method, selecting important miRNA features during the model prediction process for visual analysis of the impact of these features on CIN status at the global and local levels.

#### 3.1.1 The objective function of the classifier

In order to obtain the optimal model, the objective function needs to be minimized. We use a loss function and a regularization parameter to construct the objective function of the XGBoost model. Let *L* denote the loss function, which represents the bias of the model, and let Ω represent the regularization term, which is used to suppress model complexity. Let *n* represent the number of samples, *m* the number of features, and *K* the number of weak classifiers. The feature vector of the *i*-th sample is $x_{i}$; $y_{i}$ is the true label of $x_{i}$ ($x_{i}\in R^{m},y_{i}\in R$, *i*=1,2,......,*n*). Then the predicted value of the *i*-th sample can be defined as Eq. (1):

(1) $$\overset{ˆ}{y_{i}}=\sum_{k=1}^{K}f_{k}(x_{i}),$$

where $f_{k}$ is the *k*-th weak classifier.

The loss function can be defined as Eq. (2):

(2) $$L=\sum_{i=1}^{n}l(y_{i},\overset{ˆ}{y_{i}}),$$

where l(⋅) is a binary cross-entropy loss function.

The formula of regularization term Ω is defined as Eq. (3):

(3) $$\Omega (f_{k})=\gamma T+\frac{1}{2}\lambda \sum_{j=1}^{T}w_{j}^{2},$$

where *γT* is the regularization term of L1; $\frac{1}{2}\lambda \sum_{j=1}^{T}w_{j}^{2}$ is the regularization term of L2; and *γ* and *λ* are learning parameters that are used to prevent the overfitting problem and control the weight size of the regularization term, respectively; *T* is the number of leaf nodes; and $w_{j}$ is the node weight of the *j*-th leaf.

Thereby, the objective function of the XGBoost model can be defined as Eq. (4):

(4) $$Obj=\sum_{i=1}^{n}l(y_{i},\overset{ˆ}{y_{i}})+\sum_{k=1}^{K}\Omega (f_{k}).$$

The objective function is used to evaluate the performance of the model. The smaller the *Obj* value, the better the model's performance.

### 3.2 Feature selection strategy

One of the most commonly used methods to enhance model interpretability involves analyzing model results by examining the importance and relevance of input features. This feature selection provides good initial insight into what the model is learning and what factors might be important. XGBoost provides three methods for calculating feature importance: weight-based, gain-based, and coverage-based methods.

#### 3.2.1 Weight-based feature selection

Weight-based feature importance is determined by the frequency of a feature being used as a split point across all trees, calculated as the cumulative count of its appearances in all decision-tree splits. During the training process, XGBoost assigns a weight to each feature, indicating the number of times the feature is used in all trees. A higher weight assigned to a feature signifies that it has greater importance in the model. Let $weight_{i}$ be the importance of the *i*-th feature, which is defined as Eq. (5):

(5) $$weight_{i}=\sum_{j=1}^{T}I(f_{j}=feature_{i}),$$

where *T* is the number of decision trees, $f_{j}$ is the feature selected by the *j*-th decision tree, $feature_{i}$ is the *i*-th feature, and I(⋅) is the indicator function. When $f_{j}=feature_{i}$, the value of the indicator function is 1; otherwise, the value of the indicator function is 0.

#### 3.2.2 Gain-based feature selection

Gain-based feature importance is calculated based on the average information gain obtained by using the feature for splitting across all trees. It quantifies the average enhancement in model prediction performance that can be attributed to the feature's usage in the decision tree. This feature selection method effectively captures and quantifies the contribution of each feature. Let $gain_{i}$ be the importance of the *i*-th feature, which is defined as Eq. (6):

(6) $$gain_{i}=\frac{1}{2}\frac{\sum_{j=1}^{T}\sum_{n=1}^{N(j)}I(\beta (j,n)=i)(\frac{G_{\gamma (j,n,L)}^{2}}{H_{\gamma (j,n,L)}+\lambda }+\frac{G_{\gamma (j,n,R)}^{2}}{H_{\gamma (j,n,R)}+\lambda }-\frac{G_{\gamma (j,n)}^{2}}{H_{\gamma (j,n)}+\lambda })}{\sum_{j=1}^{T}\sum_{n=1}^{N(j)}I(\beta (j,n)=i)},$$

where N(j) is the number of non-leaf nodes of the *j*-th decision tree, β(j,n) is the splitting feature of the *n*-th non-leaf node of the *j*-th tree, and I(⋅) is the indicator function. $G_{\gamma (j,n,L)}$ and $H_{\gamma (j,n,L)}$ are the sum of the first-order derivative and the sum of the second-order derivative, respectively, on the left node of the *n*-th non-leaf node of the *j*-th tree. Similarly, $G_{\gamma (j,n,R)}$ and $H_{\gamma (j,n,R)}$ are the sum of the first-order derivative and the sum of the second-order derivative, respectively, on the right node of the *n*-th non-leaf node of the *j*-th tree.

#### 3.2.3 Coverage-based feature selection

Coverage-based feature importance is calculated according to the average coverage of a feature when it is used across all trees. It can be defined as the average sum of the second-order derivatives of the corresponding samples when a feature is used as a splitting feature. The cover value increases as the splitting takes place closer to the root node, indicating greater importance. Let $cover_{i}$ be the importance of the *i*-th feature, which is defined as Eq. (7):

(7) $$cover_{i}=\frac{\sum_{j=1}^{T}\sum_{n=1}^{N(j)}I(\beta (j,n)=i)H_{\gamma (j,n)}}{\sum_{j=1}^{T}\sum_{n=1}^{N(j)}I(\beta (j,n)=i)},$$

where $H_{\gamma (j,n)}$ is the sum of the second-order derivatives of all samples associated with the *n*-th non-leaf node of the *j*-th tree.

### 3.3 The SHAP method

Although the three methods mentioned above can achieve the aim of selecting important features, they primarily focus on the global interpretability of the model and ignore the aspect of local interpretability; thus, they fail to provide a detailed analysis of how input features participate in the model's prediction process. In the present study, we use the SHAP method for selecting important features. The SHAP method is a comprehensive model interpretability approach that is capable of addressing both global and local interpretations. It belongs to the category of model post-interpretation methods, and its core idea is to calculate the marginal contributions of features to the model's output. This calculation allows us to explain the predictions of an otherwise “black box model” at both global and local levels. From a single-sample perspective, the SHAP method provides insight into the potential relationships between the predicted values and specific features. Drawing on Shapley value theory and game theory [36], the SHAP method treats the Shapley value as an additive feature attribution approach. It constructs an interpretable model that considers all features as “contributors” to the prediction results. By calculating the SHAP value for each feature, the SHAP method can determine the influence of each feature on the overall prediction result, thus enabling a comprehensive interpretation of the overall prediction outcome.

For each input sample, the model generates a predicted value $y^{\prime }$. In contrast to linear models, the SHAP method applies a log odds ratio conversion to its predicted values when interpreting the XGBoost model. The formula is defined as Eq. (8):

(8) $$\ln\frac{y^{\prime }}{1-y^{\prime }}=\phi_{0}+\sum_{i=1}^{\left|N\right|}\phi_{i}^{shap},$$

where $\phi_{0}$ is the predicted mean value of all training samples, $\phi_{i}^{shap}$ is the contribution of feature *i* to the final prediction result, and |N| is the number of features in the input feature set.

The formula for calculation of $\phi_{i}^{shap}$ is defined as Eq. (9):

(9) $$\phi_{i}^{shap}=\sum_{S\subseteq N\setminus \{i\}}\frac{|S|!(|N|-|S|-1)!}{|N|!}[f(S\cup \{i\})-f(S)],$$

where N∖{i} is the set of all input features except feature *i*, *S* is a feature subset of N∖{i}, and f(S) is the model's predicted value for input feature subset *S*. The f(⋅) used in this work is the XGBoost model.

### 3.4 Evaluation metrics

In this study we use sensitivity (Sen), specificity (Spe), accuracy (Acc), precision (Pre), and F1-score to evaluate the comparative performance of various models. The formulas used to calculated these metrics are as Eqs. (10)-(14):

(10) $$Sen=\frac{TP}{TP+FN},$$

(11) $$Spe=\frac{TN}{TN+FP},$$

(12) $$Acc=\frac{TP+TN}{TP+TN+FP+FN},$$

(13) $$Pre=\frac{TP}{TP+FP},$$

(14) $$F1-score=\frac{2\times Pre\times Sen}{Pre+Sen},$$

where TP (true positive) represents the number of samples correctly predicted as low CIN; FP (false positive) represents the number of samples incorrectly predicted as low CIN; TN (true negative) represents the number of samples correctly predicted as high CIN; and FN (false negative) represents the number of samples incorrectly predicted as high CIN.

## 4 Materials

### 4.1 Dataset

We collated breast cancer data (TCGA-BRCA dataset) from The Cancer Genome Atlas (TCGA), a joint project funded by the National Institutes of Health and the National Cancer Institute. The TCGA-BRCA dataset mainly includes infiltrating duct carcinoma and lobular carcinoma. We downloaded clinical information, CNV data, and miRNA sequencing data of 1098 breast cancer patients from the TCGA database^1^ and calculated the CIN status of the patients based on the CNV data [37]. Following TCGA code specifications, only the miRNA and clinical features corresponding to samples with “01A” in the 14-th to 16-th positions of the sample name were retained. Finally, our dataset for the follow-up study consisted of 979 patients and 385 features, comprising 13 clinical features and 372 miRNA features.

### 4.2 Data preprocessing

We labeled CIN status by calculating the fraction of genomic alterations (FGA) from CNV data, an approach inspired by [37]. Samples with FGA greater than 0.3 were labeled as high CIN status and assigned a value of 1, whereas samples with FGA lower than 0.3 were labeled as low CIN status and assigned a value of 0. In clinical studies, high CIN status is strongly correlated with poor patient prognosis, whereas low CIN status suggests a better prognosis. The distribution of samples is shown in Table 1.

**Table 1:** Distribution of samples.

Subgroup Type	Number
Samples with high CIN	462
Samples with low CIN	517
All samples	979

Differential expression analysis was conducted on the downloaded breast cancer miRNA data using the TCGAbiolinks [38] and edgeR packages in the R software. Using a false discovery rate less than 0.01 and an absolute value of log_2_ fold change greater than 1 as thresholds, a total of 372 miRNA features with significant expression differences between normal and diseased samples were identified. To ensure data quality, we eliminated invalid features with a higher proportion of null values in the clinical data and randomly filled the remaining missing values. Table 2 shows the 13 clinical features used in the experiment.

**Table 2:** Clinical features.

Clinical features	Meaning of representation
ajcc_pathologic_stage	The specific stage of the patient's cancer defined by the combination of the three indicators of TNM based on AJCC staging criteria.
days_to_last_follow_up	Time interval from the date of last followup to the date of initial pathologic diagnosis, represented as a calculated number of days.
primary_diagnosis	Type of disease initially diagnosed.
age_at_diagnosis	Time interval from the individual's date of birth to the date of initial pathologic diagnosis, represented as a calculated number of days.
year_of_diagnosis	The year in which the disease was first diagnosed.
prior_malignancy	Whether there was any previous malignancy.
vital_status	The survival status, alive or dead.
gender	Text designations that identify gender.
race	Text for reporting information about race.
age_at_index	Age at which the disease was first diagnosed.
year_of_birth	The year of birth of the patient.
treatments_pharmaceutical_treatment_or_therapy	Received drug treatment or not.
treatments_radiation_treatment_or_therapy	Received chemotherapy treatment or not.

## 5 Experiments and results

### 5.1 Experimental settings

All experiments in this study were conducted on the Python and R platforms. The XGBoost model's main parameters were initialized as follows: learning_rate set to 0.3, n_estimators set to 100, max_depth set to 6, objective set to “binary:logistic.” We used a tree model as the base classifier for XGBoost. All experiments were conducted using the leave-out method and run on a computer with a 12-th Gen Intel(R) Core(TM) i5-12500H CPU and 16 GB running memory.

### 5.2 Comparison with other classification methods

To verify the performance of our proposed method, we compared it with other classifiers on the same dataset: decision tree (DT) [39], SVM [40], RF [41], and AdaBoost [42]. Among them, DT and SVM belong to non-ensemble learning methods, while RF and Adaboost belong to ensemble learning methods. To ensure a fair comparison, we selected 80% of the data as the training set and the remaining 20% as the test set. The classification results obtained with the five classifiers are shown in Table 3. As shown in Table 3, compared with other classifiers, our proposed method achieved the highest accuracy of 78.57%. DT and SVM had the poorest performance. This may be influenced by the size of the data and the complex relationship between features, tending towards local optima. RF achieved an accuracy of 73.47%, which was 2.04% lower than that of AdaBoost. AdaBoost ranked second among the five classifiers. RF sometimes tends to choose features with more categories, and Adaboost is sensitive to outliers in the data, making the model prone to overfitting. This may be the reason for the poor performance of these two classifiers on our experimental data. In addition, our proposed method achieved the highest sensitivity, specificity, precision, and F1-score among the five methods. We also observed that the overall performance of ensemble methods (RF, AdaBoost, and XGBoost) surpassed that of non-ensemble methods (DT and SVM). This proves that compared to a single learner, the ensemble method of integrating multiple learners through optimization strategies significantly improves generalization performance.

**Table 3:** Comparison results of the five classifiers.

	Acc	Sen	Spe	Pre	F1-score
DT [39]	61.73%	67%	55%	66%	67%
SVM [40]	67.86%	66%	70%	75%	70%
RF [41]	73.47%	73%	77%	81%	77%
AdaBoost [42]	75.51%	73%	79%	82%	77%
GL_XGBoost	**78.57%**	**76%**	**82%**	**85%**	**80%**

**Figure 2:**
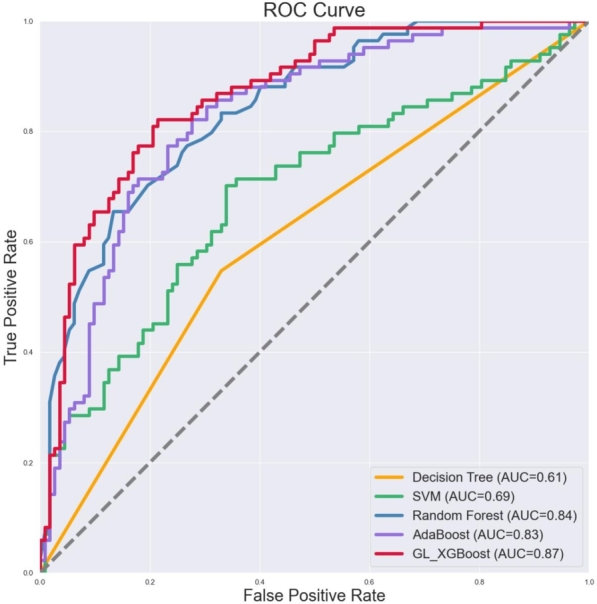
ROC curves obtained by five classifiers.

**Figure 3:**
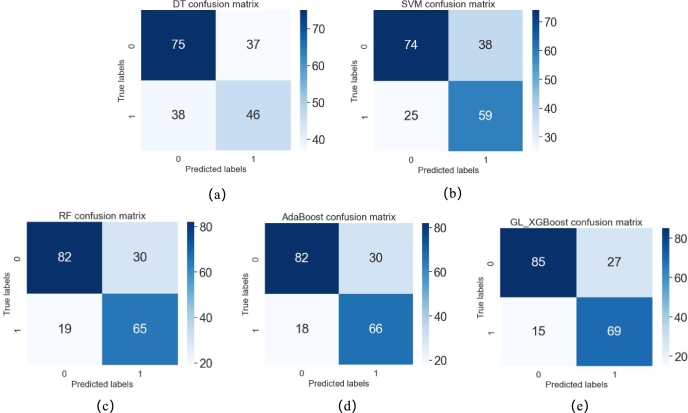
Confusion matrices obtained by five classifiers, which are DT(a), SVM(b), RF(c), AdaBoost(d) and GL_XGBoost(e) respectively.

We also evaluated these classifiers using confusion matrices and receiver operating characteristic curves. As shown in Fig. 2, the AUC values for DT, SVM, RF, AdaBoost, and GL_XGBoost on the TCGA-BRCA dataset were 0.61, 0.69, 0.84, 0.83, and 0.87, respectively. The AUC of GL_XGBoost was higher than those of the other four classifiers, particularly surpassing DT and SVM. Fig. 3 shows the confusion matrices for the five classifiers, where the vertical axis represents the true labels of the test samples and the horizontal axis represents the predicted labels. From top to bottom and from left to right, the four values in the confusion matrix represent TP, FN, FP, and TN, respectively. As shown in Fig. 3, GL_XGBoost misclassified the fewest samples, among which 15 samples with high CIN were wrongly predicted to be low CIN, and 27 samples with low CIN were wrongly predicted to be high CIN. These misclassifications accounted for 21.4% of all the tested samples. Although our classifier mistakenly predicted 27 samples with low CIN status as having high CIN status, the risk of high CIN can be excluded through examination in clinical diagnosis, making the model more sensitive and specific. As shown in Table 3, the sensitivity and specificity of GL_XGBoost were 76% and 82%, respectively, indicating good performance on the dataset used in this work.

### 5.3 Comparison of feature selection strategies

**Figure 4:**
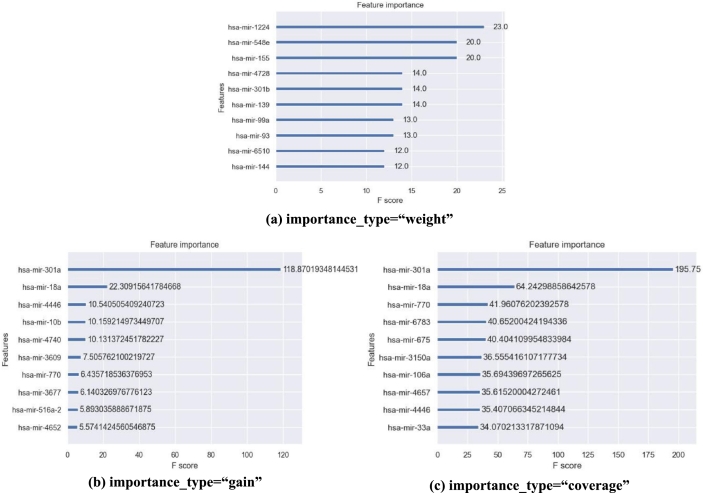
Comparison of three feature selection strategies of XGBoost model.

XGBoost provides three default feature selection strategies: weight-based, gain-based, and coverage-based. To compare the differences among these three strategies in XGBoost, we determined the top 10 important features selected by each strategy (Fig. 4). Specifically, Fig. 4 (a), (b), and (c) show the top 10 important miRNA features obtained based on weight, gain, and coverage, respectively. It is worth noting that the three feature selection results obtained were inconsistent. None of the features obtained via the weight-based strategy were selected by the other two selection strategies, whereas three of the features obtained based on gain and those based on coverage were the same. In the weight-based strategy, *hsa-mir-1224* contributed the most to the model prediction process, with an importance score of 23.0 (Fig. 4 (a)). As shown in Fig. 4 (b) and (c), *hsa-mir-301a* had the greatest impact on the predictive performance of the model. However, *hsa-mir-4446* had different effects on performance according to the two selection strategies. In the gain-based strategy, the contribution of *hsa-mir-4446* to the model prediction process ranked third, whereas in the coverage-based strategy, it ranked ninth.

The reason for these inconsistent results was that the weight-based method only considers the frequency of feature occurrences within the model. The gain-based method considers the contribution of feature splitting to model accuracy, but it may be influenced by factors such as the depth of the tree. The coverage-based method takes into account the number of samples covered but may lead to overfitting for features of high cardinality type. Strobl et al. [43] also showed that the default feature selection strategy of the model would favor continuous or high cardinality variables. Moreover, these selection strategies can only calculate feature importance from a global perspective, which is not conducive to further exploring the impact of miRNA features on CIN status.

**Figure 5:**
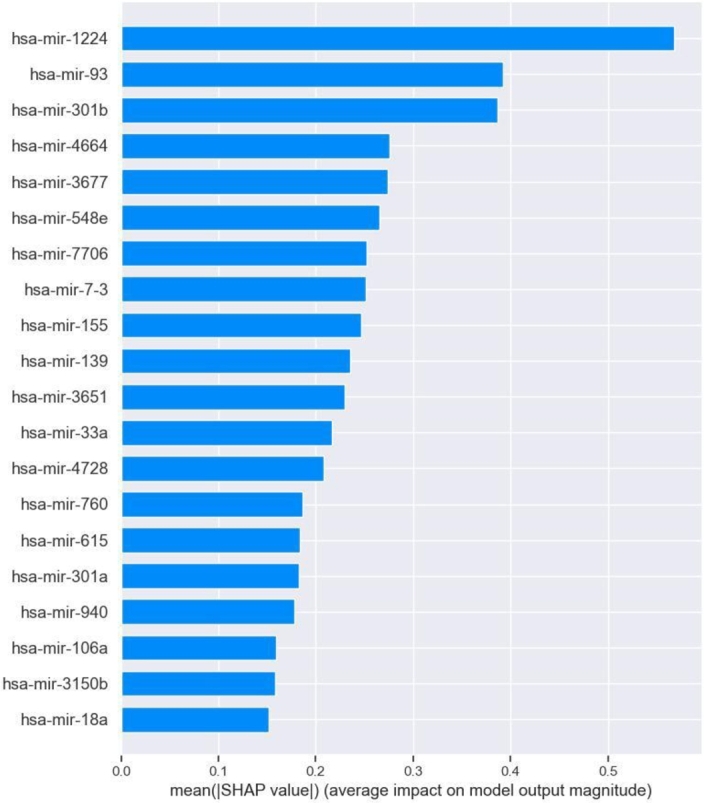
Feature importance obtained by SHAP method.

**Figure 6:**
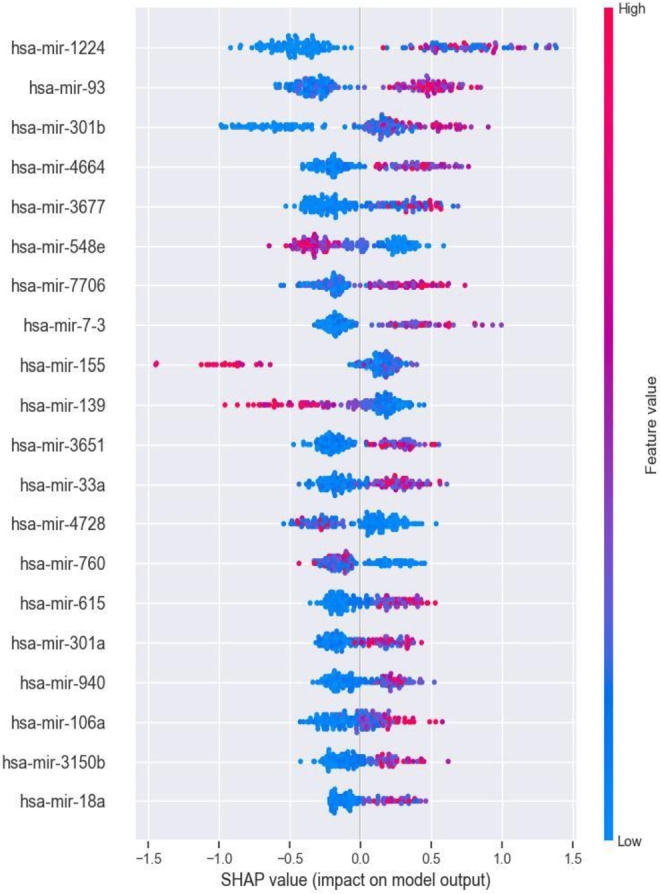
The relationships between the feature values of the top 20 important miRNA features and their SHAP values for all samples in the XGBoost model prediction process.

At present, most of the methods applied in the field of assisted medical diagnosis belong to the “black box model”. Despite achieving good results, there are still challenges in helping healthcare professionals understand the decisions made by the model. In this study, we adopted SHAP as the feature selection and interpretability method. The SHAP method can not only visualize the prediction process of a single sample but also reflect the contribution of a single feature or multiple features to the prediction results. It can interpret the model at global and local levels, which is more in line with the requirements of clinical diagnosis [44]. To further demonstrate the advantages of the SHAP method, we demonstrated the importance and contribution of features in the XGBoost model prediction process from a global perspective (Figure 5, Figure 6). We added and averaged the absolute SHAP values of all features and then sorted them in descending order. Fig. 5 shows the top 20 most important features. The ordinate represents the feature name, and the abscissa represents the average absolute SHAP value of the corresponding feature, which indicates the change in logarithmic hazard ratio caused by the feature in the model output. Among these features, *hsa-mir-1224* had the strongest influence on CIN status, making it the most important feature.

Fig. 5 shows only the overall influence of features on the model's output, whereas the summary plot provides a more detailed understanding of how these features affect the model's predictions. Fig. 6 provides valuable insight regarding the relationships between the feature values of the top 20 most important miRNA features and their corresponding SHAP values across all samples. The abscissa represents the SHAP value and the dots represent the samples. The density of the dots reflects the number of samples, with a denser concentration indicating a higher sample count. The red dots represent high feature values for a given sample, whereas the blue dots represent low feature values.

As shown in Fig. 6, when the *hsa-mir-1224* feature values were small for the majority of samples, the corresponding SHAP values were less than 0. This means that the model is more likely to predict a low CIN status for such a sample. Recent studies have demonstrated the diagnostic potential of mature *miR-1224*, derived from the precursor miRNA *mir-1224*, in gastric cancer and acute liver failure patients [45], [46]. However, not all features are as involved in the prediction process of the model as *hsa-mir-1224*. For example, *hsa-mir-548e*, *hsa-mir-155*, and *hsa-mir-139* had notably low SHAP values at higher feature values. When the feature value of *hsa-mir-139* is small, the model may yield a higher predicted value for the corresponding sample. Such a sample would be predicted to have a high CIN status, potentially leading to a poorer prognosis; this is consistent with the findings of [47]. Downregulation of mature *miR-139*, processed from the precursor miRNA *hsa-mir-139*, has been linked to aggressive tumor behavior and progression of breast cancer.

### 5.4 Visual analysis

**Figure 7:**
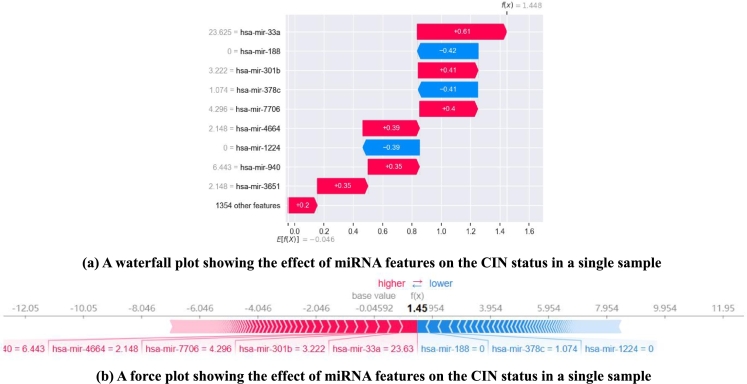
Single predictive interpretation for a patient with id “TCGA-AN-A0FZ”.

**Figure 8:**
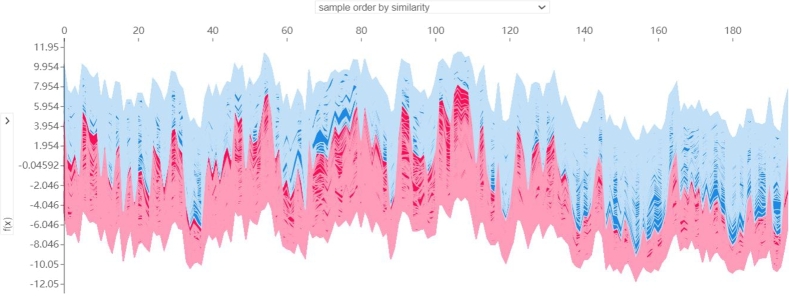
Overall visual analysis, interpreting the classification predictions of all samples in the test set.

To further analyze the relationship between CIN status and miRNA features, we conducted visual analyses of single samples and the entire sample set (Figure 7, Figure 8, respectively).

Fig. 7 shows the prediction visualization for the first sample from the test set (patient ID: TCGA-AN-A0FZ) as a waterfall plot and a force plot. In Fig. 7(a), the vertical axis represents the miRNA features and their corresponding values, whereas the horizontal axis shows the contribution of these features to the sample's prediction process, expressed as SHAP values. The final predicted value for this sample is denoted as f(x). In a nonlinear model, E[f(x)] is the model's predicted value for the mean feature value, which is the same as the base value in Fig. 7(b). Here, we use red and blue, respectively, to represent the positive and negative effects of features on CIN status. As shown in Fig. 7, the predicted value for this sample was 1.448, which was higher than E[f(x)], indicating a high CIN status. Clinical diagnosis data confirmed that the patient had been diagnosed with stage IIIA breast cancer, indicating a locally advanced stage of the disease accompanied by a high CIN status. In Fig. 7 (a) and (b), features such as *hsa-mir-33a*, *hsa-mir-301b*, and *hsa-mir-7706* correspond to the red category, indicating an increased risk of high CIN status in this sample. Conversely, features such as *hsa-mir-188*, *hsa-mir-378c*, and *hsa-mir-1224* correspond to the blue category, indicating that they weaken the risk of elevated CIN status. Among them, *hsa-mir-33a* exhibited the highest SHAP value at +0.61, implying a strong association with the CIN status of the sample. These features corresponding to the red category were also recognized by our model (Fig. 5).

Furthermore, we performed an overall visualization of all samples to gain a comprehensive understanding. The visualization results are shown in Fig. 8. We clustered the samples according to the similarity of their predicted model output values. Red and blue colors, respectively, indicate the positive and negative effects of miRNA features on CIN status. The horizontal axis represents the samples in the test set, whereas the vertical axis represents the corresponding predicted output values. We randomly observed samples from the 100-th to the 110-th position in the test set and found that the feature *hsa-mir-1224* emerged as a crucial contributor to the prediction of nearly every sample. In addition, *hsa-mir-93*, *hsa-mir-301b*, *hsa-mir-3677*, and *hsa-mir-4664* also participated in the prediction process for a large number of samples, consistent with the findings presented in Fig. 5. Notably, we also found that samples with CIN scores around the threshold of 0.3 were susceptible to false predictions. For instance, the CIN score for patient TCGA-AO-A0JI was 0.2724, indicating a low CIN status, but our model incorrectly predicted a high CIN status for this sample. Conversely, the CIN score for patient TCGA-C8-A26X was 0.3260, indicating a high CIN status, but our model incorrectly predicted a low CIN status for this sample. This may have been owing to insufficient sensitivity of our model, indicating that further investigation and refinement are warranted.

**Figure 9:**
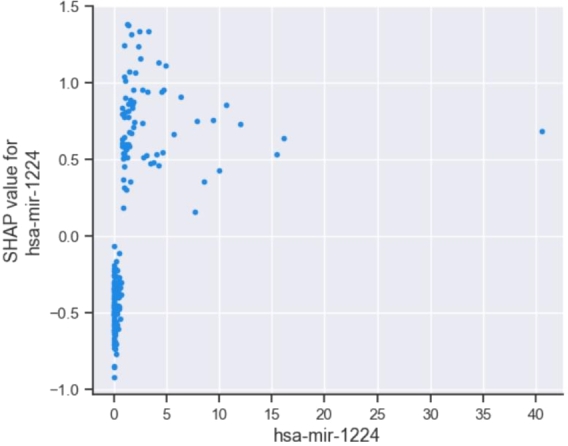
Single feature dependence plot of *hsa-mir-1224*.

**Figure 10:**
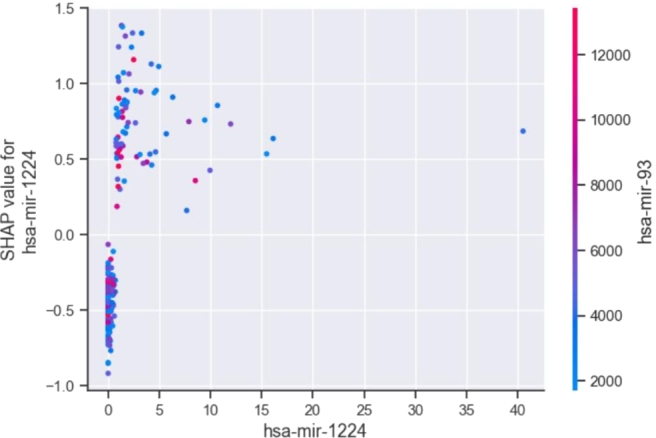
Dual feature interaction of *hsa-mir-1224* and *hsa-mir-93*.

To further analyze the interpretability advantage of the SHAP method from a local perspective, we visualized how single and multiple features behaved in the model's prediction process (Figure 9, Figure 10, respectively). In Fig. 9, the horizontal axis represents the feature values, whereas the vertical axis shows the corresponding SHAP values, and each point on the graph corresponds to a sample. Taking the feature *hsa-mir-1224* as an example, we observe that when its value is greater than 2, it increases the risk of high CIN status for the sample. When the feature value ranged from 0 to 2, it exhibits a dual effect, which may cause the model to predict a lower CIN status or a higher CIN status for the sample. Next, to further explore the correlation between miRNA features and CIN status, we demonstrated the visualization effect of the SHAP method on the interaction between features. Fig. 10 illustrates the interaction between *hsa-mir-1224* and *hsa-mir-93*. The horizontal axis and the right vertical axis represent the feature values of these two features, respectively. The combination of *hsa-mir-1224* and *hsa-mir-93* was positively correlated with CIN status. When both feature values were at a lower level, the sample was more likely to have a low CIN status. As the feature values of *hsa-mir-1224* and *hsa-mir-93* increased, the sample's CIN status tended to be high.

## 6 Discussion

CIN serves as the basis of many diseases and is associated with tumor aggressiveness, drug resistance, and poor patient prognosis in particular. In this study, we propose a CIN status classification method called GL_XGBoost that combines global and local interpretability and study the relationships between miRNA features of breast cancer patients and their CIN status. Our proposed method achieved the best classification performance among five comparative methods. In addition, due to the inconsistent results of the built-in feature filtering method in XGBoost classifier, we have re-selected two methods for calculating feature importance: permutation importance (PI) and SHAP. Through experimental testing, we found that the PI method tends to be related to other features and/or features with multiple categories, and is not suitable for interpreting tree based models [48]. Finally, we decided to use the TreeSHAP method specifically designed for tree models for feature selecting. We used the SHAP method to analyze the model's prediction process and interpret the experimental results from multiple perspectives. Global summary plots (Figure 5, Figure 6) were constructed to explain the importance of diverse miRNA features in predicting the CIN status of breast cancer patients. Moreover, a local interpretation force plot and dependency plot were used to investigate the impact of different miRNA features on the CIN status of a single sample (Fig. 7) and analyze how varying feature values affect the prediction process of the model (Fig. 9). In fact, we have also attempted to use partial dependency plots (PDP) to analyze the impact of input features on prediction results. However, due to the specificity of the experimental data, the result plots cannot effectively demonstrate the impact of miRNA features on the patient's CIN status. Meanwhile, the PDP method can only explain the prediction process of the model from the local feature level, making it difficult to comprehensively and reasonably analyze the process of predicting CIN status based on miRNA features. Rich visual analysis enables researchers to intuitively understand a model's prediction behavior and the relevance of different features, and, in this case, the interplay between feature combinations and CIN status. Consequently, our proposed approach significantly enhances the interpretability of the model.

The relationship between miRNAs and CIN has been an area of great research interest. miRNAs are known to regulate gene expression, affecting processes including cell growth, differentiation, and apoptosis, all of which are closely related to chromosome stability. In this study, we conducted a differential expression analysis of miRNA data obtained from breast cancer patients. Our proposed method predicted and explained the relationships between miRNA features and CIN status, contributing to a better understanding of CIN development. Moreover, we identified miRNA features that had the greatest impact on CIN status prediction, with those features having a higher degree of influence being more strongly associated with CIN occurrence. However, how miRNA features specifically affect chromosomal abnormalities needs to be explored from a more specialized field of biology. Due to limitations in expertise and resources, our current research cannot explain this issue in detail. Exploring the relationships between miRNAs and CIN could enable us to identify disease-related miRNAs; select potential biomarkers for early diagnosis, assessment of prognosis, and treatment monitoring; and provide a better theoretical foundation for miRNAs as therapeutic targets. In summary, our proposed method demonstrates advantages in CIN prediction and contributes to our understanding of the relationship between miRNA features and CIN. However, the present study was limited by its use of single-type miRNA data to explore the CIN status of patients, as the correlations between miRNA features and CIN were not exclusive. Additional data modalities, including gene methylation and RNA sequencing, are also interconnected with the emergence of CIN. Therefore, in the future, we will continue to delve into more modal information to better understand the complexity of CIN status. Moreover, we will combine CIN with histopathological images to conduct accurate prognosis and survival analysis for breast cancer patients.

## 7 Conclusion

In this study, we investigated the CIN status of breast cancer patients using CNV data, miRNA sequencing data, and clinical data from the TCGA-BRCA dataset. We propose a CIN status classification method named GL_XGBoost, which integrates both global and local interpretability. Experimental results demonstrate the performance of our proposed method, with an accuracy of 78.57% and an AUC of 0.87, higher than those of comparative methods. Furthermore, we employed the SHAP method to interpret important features and analyze the prediction mechanism of the model. We present and analyze these important features separately. In addition, various visualizations illustrate the correlations between miRNA features and CIN, thereby explaining the “black box model” from both global and local perspectives. This study provides valuable insights for early diagnosis, prognostic assessment, and treatment monitoring of breast cancer patients and could facilitate the selection of potential biomarkers.

## Funding

This work was funded by the Henan Provincial Key Research and Promotion Projects (No. 222102310085), the Key R&D projects during the 14th Five Year Plan period (No. 2022YFD1400302), and the 10.13039/501100001809National Natural Science Foundation of China (Grant No. 82274370).

## CRediT authorship contribution statement

**Liangliang Liu:** Writing – review & editing, Project administration, Methodology, Investigation. **Pei Zhang:** Writing – original draft, Software, Resources, Methodology. **Zhihong Liu:** Validation, Data curation. **Tong Sun:** Visualization. **Hongbo Qiao:** Writing – review & editing, Investigation.

## Declaration of Competing Interest

The authors declare that they have no known competing financial interests or personal relationships that could have appeared to influence the work reported in this paper.

## Data Availability

The dataset is a public dataset that comes from The Cancer Genome Atlas (TCGA), a joint project funded by the National Institutes of Health and the National Cancer Institute.

## Footnotes

1 https://cancergenome.nih.gov/.

## References

[1] Sung H., Ferlay J., Siegel R.L., Laversanne M., Soerjomataram I., Jemal A., Bray F. (2021). Global cancer statistics 2020: globocan estimates of incidence and mortality worldwide for 36 cancers in 185 countries. CA Cancer J. Clin..

[2] Bakhoum S.F., Cantley L.C. (2018). The multifaceted role of chromosomal instability in cancer and its microenvironment. Cell.

[3] Bach D.-H., Zhang W., Sood A.K. (2019). Chromosomal instability in tumor initiation and development. Cancer Res..

[4] Salgueiro L., Buccitelli C., Rowald K., Somogyi K., Kandala S., Korbel J.O., Sotillo R. (2020). Acquisition of chromosome instability is a mechanism to evade oncogene addiction. EMBO Mol. Med..

[5] Vishwakarma R., McManus K.J. (2020). Chromosome instability; implications in cancer development, progression, and clinical outcomes. Cancers (Basel).

[6] Sansregret L., Vanhaesebroeck B., Swanton C. (2018). Determinants and clinical implications of chromosomal instability in cancer. Nat. Rev. Clin. Oncol..

[7] Walther A., Houlston R., Tomlinson I. (2008). Association between chromosomal instability and prognosis in colorectal cancer: a meta-analysis. Gut.

[8] How C., Bruce J., So J., Pintilie M., Haibe-Kains B., Hui A., Clarke B.A., Hedley D.W., Hill R.P., Milosevic M., Fyles A., Liu F.-F. (2015). Chromosomal instability as a prognostic marker in cervical cancer. BMC Cancer.

[9] Bakhoum S.F., Ngo B., Laughney A.M., Cavallo J.-A., Murphy C.J., Ly P. (2018). Chromosomal instability drives metastasis through a cytosolic dna response. Nature.

[10] Mohapatra S., Winkle M., Ton A.N., Nguyen D., Calin G.A. (2023). The role of non-coding rnas in chromosomal instability in cancer. J. Pharmacol. Exp. Ther..

[11] Liu B., Li J., Cairns M.J. (2014). Identifying mirnas, targets and functions. Brief. Bioinform..

[12] Cui S., Liao X., Ye C., Yin X., Liu M., Hong Y., Yu M., Liu Y., Liang H., Zhang C.-Y., Chen X. (2017). Ing5 suppresses breast cancer progression and is regulated by mir-24. Mol. Cancer.

[13] Naghizadeh S., Mohammadi A., Duijf P.H.G., Baradaran B., Safarzadeh E., Cho W.C.-S., Mansoori B. (2020). The role of mir-34 in cancer drug resistance. J. Cell. Physiol..

[14] Ren Z., He M., Shen T., Wang K., Meng Q., Chen X., Zhou L., Han Y., Ji C., Liu S., Fu Q. (2020). Mir-421 promotes the development of osteosarcoma by regulating mcpip1 expression. Cancer Biol. Ther..

[15] Welponer H., Tsibulak I., Wieser V., Degasper C., Shivalingaiah G., Wenzel S., Sprung S., Marth C., Hackl H., Fiegl H., Zeimet A.G. (2020). The mir-34 family and its clinical significance in ovarian cancer. J. Cancer.

[16] Liu R., Kong W., Zheng S., Yu C., Yu Y., Xu Y., Ye L., Shao Y. (2021). Prognostic significance of microrna mir-24 in cancers: a meta-analysis. Bioengineered.

[17] Smid M., Hoes M., Sieuwerts A.M., Sleijfer S., Zhang Y., Wang Y., Foekens J.A., Martens J.W.M. (2011). Patterns and incidence of chromosomal instability and their prognostic relevance in breast cancer subtypes. Breast Cancer Res. Treat..

[18] Carter S.L., Eklund A.C., Kohane I.S., Harris L.N., Szallasi Z. (2006). A signature of chromosomal instability inferred from gene expression profiles predicts clinical outcome in multiple human cancers. Nat. Genet..

[19] McClelland S.E. (2017). Role of chromosomal instability in cancer progression. Endocr.-Relat. Cancer.

[20] Vargas-Rondón N., Villegas V.E., Rondón-Lagos M. (2017). The role of chromosomal instability in cancer and therapeutic responses. Cancers (Basel).

[21] Pino M.S., Chung D.C. (2010). The chromosomal instability pathway in colon cancer. Gastroenterology.

[22] Maleki S.S., Röcken C. (2017). Chromosomal instability in gastric cancer biology. Neoplasia (New York, N.Y.).

[23] Venkatesan S., Angelova M., Puttick C., Zhai H., Caswell D.R., Lu W.-T., Dietzen M., Galanos P. (2021). Induction of apobec3 exacerbates dna replication stress and chromosomal instability in early breast and lung cancer evolution. Cancer Discov..

[24] Volinia S., Galasso M., Sana M.E., Wise T.F., Palatini J., Huebner K., Croce C.M. (2012). Breast cancer signatures for invasiveness and prognosis defined by deep sequencing of microrna. Proc. Natl. Acad. Sci. USA.

[25] Wu J., Hicks C. (2021). Breast cancer type classification using machine learning. J. Pers. Med..

[26] Podolsky M.D., Barchuk A.A., Kuznetcov V.I., Gusarova N.F., Gaidukov V.S., Tarakanov S.A. (2016). Evaluation of machine learning algorithm utilization for lung cancer classification based on gene expression levels. Asian Pac. J. Cancer Prev..

[27] Plaimas K., Eils R., König R. (2010). Identifying essential genes in bacterial metabolic networks with machine learning methods. BMC Syst. Biol..

[28] Liu L., Cheng J., Quan Q., Wu F.-X., Wang Y.-P., Wang J. (2020). A survey on u-shaped networks in medical image segmentations. Neurocomputing.

[29] Liu L., Wang Y.-P., Wang Y., Zhang P., Xiong S. (2022). An enhanced multi-modal brain graph network for classifying neuropsychiatric disorders. Med. Image Anal..

[30] Ahmed O., Brifcani A. (2019). 2019 4th Scientific International Conference Najaf (SICN).

[31] Taylor R.A., Moore C.L., Cheung K.-H., Brandt C. (2018). Predicting urinary tract infections in the emergency department with machine learning. PLoS ONE.

[32] Iwase S., Nakada T.-A., Shimada T., Oami T., Shimazui T., Takahashi N., Yamabe J., Yamao Y., Kawakami E. (2022). Prediction algorithm for icu mortality and length of stay using machine learning. Sci. Rep..

[33] Devan P., Khare N. (2020). An efficient xgboost–dnn-based classification model for network intrusion detection system. Neural Comput. Appl..

[34] Chen T., Guestrin C. (2016).

[35] Haidar A., Verma B.K., Haidar R. (2019). A swarm based optimization of the xgboost parameters. Aust. J. Intell. Inf. Process. Syst..

[36] Lundberg S.M., Lee S.-I. (2017). Proceedings of the 31st International Conference on Neural Information Processing Systems.

[37] Xu Z., Verma A., Naveed U., Bakhoum S.F., Khosravi P., Elemento O. (2021). Deep learning predicts chromosomal instability from histopathology images. iScience.

[38] Colaprico A., Silva T.C., Olsen C., Garofano L., Cava C., Garolini D., Sabedot T.S. (2016). Tcgabiolinks: an r/bioconductor package for integrative analysis of tcga data. Nucleic Acids Res..

[39] Quinlan J.R. (1986). Induction of decision trees. Mach. Learn..

[40] Cortes C., Vapnik V. (1995). Support-vector networks. Mach. Learn..

[41] Breiman L. (2001). Random forests. Mach. Learn..

[42] Freund Y., Schapire R.E. (1996). Proceedings of the Thirteenth International Conference on International Conference on Machine Learning.

[43] Strobl C., Boulesteix A.-L., Zeileis A., Hothorn T. (2007). Bias in random forest variable importance measures: illustrations, sources and a solution. BMC Bioinform..

[44] Lundberg S.M., Nair B., Vavilala M.S., Horibe M., Eisses M.J., Adams T., Liston D.E., Low D.K.-W., Newman S.-F. (2018). Explainable machine-learning predictions for the prevention of hypoxaemia during surgery. Nature Biomed. Eng..

[45] Roy S., Bantel H., Wandrer F., Schneider A.T., Gautheron J., Vucur M., Tacke F., Trautwein C., Luedde T., Roderburg C. (2017). mir-1224 inhibits cell proliferation in acute liver failure by targeting the antiapoptotic gene nfib. J. Hepatol..

[46] Han G.-D., Sun Y., Hui H.-X., Tao M.-Y., Liu Y.-Q., Zhu J. (2021). Mir-1224 acts as a prognostic biomarker and inhibits the progression of gastric cancer by targeting satb1. Front. Oncol..

[47] Dai H., Gallagher D., Schmitt S., Pessetto Z.Y., Fan F., Godwin A.K., Tawfik O. (2017). Role of mir-139 as a surrogate marker for tumor aggression in breast cancer. Hum. Pathol..

[48] Hooker G., Mentch L.K., Zhou S. (2021). Unrestricted permutation forces extrapolation: variable importance requires at least one more model, or there is no free variable importance. Stat. Comput..

